# Evolution of Mo-Rich Carbides in H13 Hot-Working Die Steel During Spheroidizing Annealing

**DOI:** 10.3390/ma19214621

**Published:** 2026-10-30

**Authors:** Kun Yi, Tianyu Li, Junwei Yin, Wenxiong Chen, Zhenyong Xie, Yanhua Xue, Xiaoke Qu, Qi Jiang, Qing Cui, Chen Chen, Linhao Tan

**Affiliations:** 1 Chengdu Advanced Metal Materials Industrial Technology Research Institute Co., Ltd., Chengdu 610300, China; 2 State Key Laboratory of Metal Materials for Marine Equipment and Their Applications, Anshan 114009, China; 3 Pangang Group Jiangyou Changcheng Special Steel Co., Ltd., Mianyang 621700, China

**Keywords:** hot-working die steel, spheroidizing annealing, carbide, precipitation

## Abstract

In this study, the evolution behavior of Mo-rich carbides in typical hot-working die steel (H13) during spheroidizing annealing at solution temperatures of 950–1150 °C was systematically investigated by means of thermodynamic calculations combined with OM, SEM, TEM, and XRD characterization, as well as V-notch Charpy impact and Rockwell hardness tests. The aim was to reveal the morphological characteristics, size distribution, and evolution mechanisms of Mo-rich carbides during spheroidizing annealing of H13 steel and to avoid the formation of large irregular M6C carbides. The results indicate that the dissolution difficulty of carbides follows the order: V-rich carbide > Mo-rich carbide ≈ Cr-rich carbide. Following low-temperature solution treatment (950 °C), the Mo-rich carbide precipitates uniformly in a dispersed manner during spheroidizing annealing. In contrast, high-temperature solution treatment (1150 °C) leads to the formation of a network structure along the grain boundaries after spheroidizing annealing. The evolution of the Mo-rich carbide is determined by a combination of thermodynamic conditions, the low diffusivity of Mo atoms and nucleation sites. Taking into account the morphology, size, distribution and spheroidization quality of the Mo-rich carbides, 1000 °C provides the most favourable combination of the investigated microstructural characteristics among the conditions examined. After quenching and triple tempering, the Rockwell hardness of all the samples lies within a narrow range of 44–46 HRC, indicating comparable strength levels. The V-notch impact energy also reaches its highest value of 18.3 J at 1000 °C (13.0 J at 1150 °C). This variation correlates closely with the grain size and the distribution of the Mo-rich carbides. This study provides a reliable theoretical basis for optimizing the post-forging heat treatment process of hot-working die steel.

## 1. Introduction

Hot-working die steel is widely used in the manufacture of dies for aluminum, magnesium, and copper alloy die casting, hot extrusion, and hot forging, among other applications, owing to its excellent hardenability, high toughness, and superior thermal fatigue resistance [1,2,3,4]. Hot-working die steel contains carbide-forming elements such as Cr, Mo, and V, which enable the precipitation of various carbides, including M_23_C_6_ (Cr-rich), Cr_7_C_3_ (Cr-rich), MC (V-rich), M_2_C (Mo-rich), and M_6_C (Mo-rich). The morphology, size, distribution, and other characteristics of these carbides exert a decisive influence on the final performance of the material [5,6,7,8,9,10].

Extensive research has been conducted on the evolution behavior of various carbides in Cr-Mo-V hot-working die steel [7,8,9,10,11,12,13,14,15,16,17,18,19]. Kang et al. investigated the effects of spheroidizing annealing temperature and cooling rate on carbides in H13 steel and found that Mo-rich carbides formed during the transformation from M_7_C_3_ to M_23_C_6_ [5]. Ding et al. reported a modified H13 steel with increased W and Mo contents and reduced Cr content, in which a large number of nanoscale Mo_2_C particles hindered dislocation rearrangement, thereby enhancing its resistance to temper softening [6]. Wang et al. controlled the pre-precipitation of VC carbides from austenite and obtained excellent ductility and toughness [17]. Gong et al. reported that Ni enhances the diffusion coefficient of Cr atoms in the face-centered cubic (fcc) Fe matrix during tempering and improves the phase transition driving force of various precipitates in Cr-Mo-V hot-working steel [18].

Among these carbides, Mo-rich carbides (such as M_2_C and M_6_C), when precipitated as nanoscale secondary carbides during tempering, can significantly enhance the precipitation strengthening effect, serving as key strengthening carbides that enable hot-working die steel to achieve secondary hardening and exhibit good high-temperature strength [5,8,19,20,21,22,23]. However, improper control of these strengthening carbides can also adversely affect material properties. When carbides become coarser or precipitate along grain boundaries to form network carbides, the toughness and thermal fatigue resistance of the material are severely degraded [5,6]. It has been reported that performing a solution treatment prior to spheroidizing annealing can dissolve the carbides, resulting in finer and more uniformly distributed carbides after spheroidizing annealing [24,25]. Nevertheless, existing studies have mostly focused on the overall quantity and distribution of carbides, while the dissolution, precipitation, and spheroidization behaviors of Mo-rich carbides during spheroidizing annealing have not yet been systematically investigated. In particular, no reports have addressed the tendency of Mo-rich phases to precipitate along grain boundaries during spheroidizing annealing after high-temperature solution treatment.

In this study, H13 hot-working die steel was selected as the research object, and different solution temperatures (950–1150 °C) were employed to obtain prior microstructures with distinct carbide characteristics. The precipitation and spheroidization behavior of Mo-rich carbides during spheroidizing annealing was systematically investigated. This work is of significant theoretical importance and engineering application value for optimizing the heat treatment process of hot-working die steel, further tailoring its microstructure, and improving the service life of dies.

## 2. Experimental Section

### 2.1. Sample Preparation

The experimental steel was prepared via electric furnace melting, electroslag remelting, high-temperature homogenization, forging, and softening annealing, resulting in a final cross-section of 800 mm × 300 mm. One test sample with a thickness of 20 mm was sectioned for experimentation, and its chemical composition was experimentally measured and is listed in Table 1.

Multiple metallographic samples with dimensions of 15 mm × 15 mm × 20 mm were sectioned for solution treatment and spheroidizing annealing, as illustrated in Figure 1. The samples were held at 950 °C, 1000 °C, 1050 °C, 1100 °C, and 1150 °C for 1 h, respectively, followed by oil quenching upon removal from the furnace to complete the solution treatment. Subsequently, the samples were held at 860 °C for 4 h, cooled to 740 °C at a rate of 30 °C/h, held at 740 °C for 8 h, then further cooled to 500 °C at 30 °C/h, and finally removed from the furnace for air cooling to complete the spheroidizing annealing process.

To investigate the precipitation and spheroidization behavior of carbides during the spheroidizing annealing, interrupted experiments were conducted on samples corresponding to solution treatment conditions ①, ③, and ⑤. Specifically, oil quenching was applied to the samples at selected stages during the spheroidizing annealing process to preserve the carbide morphologies of intermediate-state samples at different stages of spheroidizing annealing. Three states, designated as A, B, and C in Figure 1, were selected for the interrupted samples: Point A corresponds to oil quenching after holding at 860 °C for 4 h; Point B corresponds to oil quenching after further cooling to 740 °C; and point C corresponds to oil quenching after continued holding at 740 °C for 8 h.

To improve the readability and traceability of the samples throughout the different heat-treatment and characterization stages, a consistent sample designation was adopted, as summarized in Table 2. These designations are used throughout the text and the figure captions. Each sample is designated by its solution-treatment temperature (950, 1000, 1050, 1100, or 1150) followed by a state code: ST for the as-solution-treated (oil-quenched) condition, A, B, and C for the three interrupted spheroidizing-annealing points defined in Figure 1, and F for the final spheroidized condition. For example, 950-ST denotes the sample solution-treated at 950 °C, 1050-B denotes the sample solution-treated at 1050 °C and oil-quenched at point B of spheroidizing annealing, and 1150-F denotes the sample solution-treated at 1150 °C followed by complete spheroidizing annealing.

### 2.2. Microstructure Characterization

The samples were first mechanically ground using 120#, 400#, 1000#, and 1500# sandpaper in sequence, followed by mechanical polishing with a 2.5 μm diamond suspension. After cleaning with alcohol, the samples were etched with a 4% nitric acid alcohol solution, rinsed with clean water, and dried with hot air to obtain the desired metallographic samples. The microstructure was observed using a ZEISS Axio Imager A1m optical microscope (Carl Zeiss Microscopy GmbH, Germany) and a Thermo Scientific Quattro S scanning electron microscope (Thermo Fisher Scientific, USA). Secondary electron mode was employed to observe the etched microstructure, and energy-dispersive spectroscopy (EDS) equipped on the SEM was used for elemental analysis of selected microregions. To distinguish different types of carbides, backscattered electron (BSE) imaging was utilized to identify various carbide species within the microstructure. The size and number density of carbides were statistically analyzed using Image-Pro Plus software. For each condition, five fields of view were examined, covering a total statistical area of 33,715 μm^2^. For each heat-treatment condition, the five fields of view were obtained from one metallographic sample, and the whole heat-treatment and characterization procedure was repeated on at least two independently heat-treated samples to confirm the reproducibility of the statistical results. The error bars in the quantitative figures represent the standard deviation. Further microstructural characterization was performed using an F200 transmission electron microscope (TEM). Carbide identification was performed using a Bruker D8 ADVANCE X-ray diffractometer (Bruker AXS GmbH, Germany) with Cu Kα radiation, a scanning rate of 1°/min, a step size of 0.02°, and a scanning range of 20° to 120°. Thermodynamic calculations of carbide equilibria for the experimental steel were carried out using JMatPro.

### 2.3. Mechanical Property Tests

Before the impact specimens were machined, the blanks for the impact tests were subjected to the same solution treatment and spheroidizing annealing as described in Section 2.1 and were then quenched and tempered. The quenching treatment consisted of austenitizing at 1030 °C for 30 min followed by oil quenching. The tempering treatment consisted of three consecutive tempering cycles: the first tempering at 600 °C for 2 h, the second tempering at 610 °C for 2 h, and the third tempering at 590 °C for 2 h, each followed by air cooling.

V-notch Charpy impact specimens with dimensions of 10 mm × 10 mm × 55 mm were machined from the quenched-and-tempered blanks. The V-notch, with a depth of 2 mm and an included angle of 45°, was introduced after the heat treatment. The impact tests were conducted at room temperature using a pendulum impact testing machine, and the absorbed energy (KV2) was recorded. For each condition, three specimens were tested and the average value was reported. In addition, the Rockwell hardness (HRC) of the quenched-and-tempered samples was measured on a Rockwell hardness tester.

## 3. Results and Discussion

### 3.1. Thermodynamic Calculations

The equilibrium carbide diagram of the experimental steel was calculated using JMatPro thermodynamic software, and the results are presented in Figure 2. According to the calculation results, solidification of the experimental steel begins at 1481 °C. Below 1395 °C, the liquid carbide completely transforms into a solid carbide, and the resultant microstructure consists of single-carbide austenite. Upon completion of solidification, the MC carbide (V-rich carbide) starts to precipitate when the temperature drops to 1095 °C, followed by the precipitation of the M_2_C carbide (Mo-rich carbide) at 936 °C. The M_7_C_3_ carbide (Cr-rich carbide) begins to precipitate at 909 °C. When the temperature decreases to 808 °C, the M_7_C_3_ carbide gradually transforms into the M_23_C_6_ carbide (Cr-rich carbide), accompanied by extensive precipitation of the M_23_C_6_ carbide.

### 3.2. Primary Microstructure

The experimental steel was in the as-forged and softening-annealed condition (860 °C × 10 h), and its microstructure is shown in Figure 3. As can be seen from Figure 3a, a large number of chain carbides existed in the as-forged microstructure. Based on atomic number contrast, different types of carbides could be distinguished in the backscattered electron (BSE) images (Figure 3b–d): bright white particles were identified as Mo-rich carbides (indicated by red arrows in Figure 3), gray particles as Cr-rich carbides (indicated by yellow arrows), and black particles as V-rich carbides (indicated by blue arrows). The chain carbides consisted predominantly of Cr-rich and Mo-rich carbides. Softening annealing alone after forging failed to sufficiently alleviate the carbide segregation.

### 3.3. Effect of Solution Temperature on the Spheroidized Microstructure

Figure 4 shows the backscattered electron (BSE) images of the samples in the as-solution-treated (ST) state. In the 950-ST sample, a considerable amount of Mo-rich, V-rich, and Cr-rich carbides remained undissolved, although the particles were relatively fine. In the 1000-ST sample, the degree of carbide dissolution increased significantly, and the amounts of Cr-rich and Mo-rich carbides in the matrix decreased substantially. In the 1050-ST sample, both Cr-rich and Mo-rich carbides were completely dissolved. In contrast, V-rich carbides required the highest temperature for complete dissolution. Even in the 1150-ST sample, a very small number of nanoscale V-rich carbides still persisted. This result is generally consistent with the thermodynamic carbide diagram shown in Figure 2, i.e., the dissolution temperature follows the order: V-rich carbides > Mo-rich carbides ≈ Cr-rich carbides. Meanwhile, in the 1100-ST and 1150-ST samples, the reduced number of undissolved carbides weakened their pinning effect on grain boundaries, and the accelerated grain boundary migration rate at elevated temperatures led to a certain degree of grain coarsening.

The solution treatment influenced the carbide distribution prior to spheroidizing annealing, leading to distinct microstructural morphologies after spheroidizing annealing, as shown in Figure 5. In the 950-F and 1000-F samples, the carbides were distributed relatively uniformly. In the 1050-F, 1100-F, and 1150-F samples, carbide agglomeration occurred, and network carbides formed along grain boundaries. Higher solution temperatures resulted in a reduced quantity, increased size, and significantly enhanced tendency for grain boundary precipitation of Mo-rich carbides in the F-state samples, with their morphology transitioning from granular to elongated or irregular shapes. In contrast to Mo-rich carbides, V-rich and Cr-rich carbides were finely dispersed as small granular particles within the ferrite matrix.

Figure 6 presents the XRD patterns of the samples after solution treatment at different temperatures followed by spheroidizing annealing. The Cr-rich M_23_C_6_ and M_7_C_3_ carbides, V-rich MC carbides, and Mo-rich M6C carbides are labeled in the figure. With increasing solution temperature, the diffraction M_6_C peaks of the F-state samples progressively weakened, indicating that the fraction of Mo-rich carbides gradually decreased. It should be noted that the carbide types given in Figure 2 are the equilibrium phases predicted by the thermodynamic calculation, in which M_2_C is the Mo-rich carbide predicted to precipitate at 936 °C. The Mo-rich carbide experimentally identified by XRD in the spheroidizing-annealed samples, however, is of the M_6_C type (Figure 6). Therefore, the general term “Mo-rich carbides” is used throughout this paper, and the specific carbide type is stated only where it is unambiguous: M_2_C refers to the phase predicted by the thermodynamic calculation, whereas M_6_C refers to the phase identified experimentally.

The size distribution of Mo-rich carbides in the experimental steel under different solution conditions during post-forging heat treatment, with the corresponding statistical results, is shown in Figure 7. As the solution temperature increased, the amount of dissolved carbides gradually increased, resulting in a continuous decrease in both the number of carbides and their area fraction. At a solution temperature of 1150 °C, nearly all carbides were dissolved (see Supplementary Material Figure S1). Combined with Figure 4e, the residual carbides were identified as V-rich MC carbides. However, the number of Mo-rich carbides decreased sharply when the solution temperature rose from 950 °C to 1000 °C, and they were completely dissolved into the matrix when the temperature exceeded 1050 °C. The total carbide number density and area fraction of the F-state samples exhibited an increasing trend with rising solution temperature. The area fraction of Mo-rich carbides in the F-state samples also decreased with increasing solution temperature. The number density of Mo-rich carbides first increased as the solution temperature rose from 950 °C to 1000 °C, and then decreased with further increases in solution temperature.

Figure 7e presents the average size and *d*_max_/*d*_min_ ratio (the ratio of the longest dimension to the shortest dimension of individual precipitate particles) of Mo-rich carbides and all carbides in the experimental steel after spheroidizing annealing. The solution temperature had little effect on the average carbide size (approximately 0.2 μm) and *d*_max_/*d*_min_ ratio (approximately 1.4) after spheroidizing annealing (see Supplementary Material Figure S1). In contrast, the solution temperature exerted a significant influence on the average size and *d*_max_/*d*_min_ ratio of Mo-rich carbides after spheroidizing annealing. With increasing solution temperature, both the average size and the d_max_/d_min_ ratio of Mo-rich carbides in the F-state samples increased continuously, indicating that as the Mo-rich carbides grew larger, their roundness decreased markedly, resulting in irregular or chain-like morphologies (Figure 5d–f).

### 3.4. Evolution of Mo-Rich Carbides During Spheroidizing Annealing

To further clarify the precipitation, growth, and spheroidization behavior of Mo-rich carbides at various stages of spheroidizing annealing, this section systematically analyzes the morphology, size distribution, and number density of Mo-rich carbides in the experimental steel subjected to three representative solution temperatures (950 °C, 1050 °C, and 1150 °C) during the spheroidizing annealing process (Points A, B, C, and F). Figure 8 shows the evolution of Mo-rich carbides in the samples during spheroidizing annealing after solution treatment at 950 °C, 1050 °C, and 1150 °C. To quantitatively analyze the size evolution of Mo-rich carbides during spheroidizing annealing, the size distributions of Mo-rich carbides at each stage of spheroidizing annealing were statistically analyzed for the three representative solution temperatures, and the results are presented in Figure 9.

The Mo-rich carbides in the 950-ST sample maintained a spherical morphology throughout the spheroidizing annealing process (Figure 8(b1,c1,d1,e1,f1)). The residual Mo-rich carbides in the 950-ST sample were mainly concentrated in the size range of 0.1–0.3 μm. After holding at 860 °C for 4 h (the 950-A sample, Figure 8(c1)), the number density of Mo-rich carbides increased slightly, while the area fraction of Mo-rich carbides increased notably, with the size mainly distributed in the range of 0.2–0.5 μm. Upon slow cooling from 860 °C to 740 °C (the 950-B sample, Figure 8(d1)), both the number density and area fraction of Mo-rich carbides increased considerably, with the size mainly distributed in the range of 0.2–0.6 μm. After further holding at 740 °C for 8 h (the 950-C sample, Figure 8(e1)), the number density of Mo-rich carbides decreased slightly while the area fraction continued to increase, indicating that particle growth dominated during this stage. The size was mainly distributed in the range of 0.2–0.7 μm. The transition from Point C to the final spheroidized condition (Figure 8(e1,f1)) involved a slow cooling process. A small number of fine Mo-rich carbides (0.1–0.3 μm) precipitated, resulting in a slight increase in both number density and area fraction, with the size mainly distributed in the range of 0.2–0.7 μm. The average size of Mo-rich carbides in the 950-ST sample increased gradually during spheroidizing annealing, from 0.23 μm in the ST state to 0.42 μm in the 950-F sample. Moreover, the *d*_max_/*d*_min_ ratio remained below 1.6 throughout the process, indicating that the particles maintained a near-spherical shape with a high degree of spheroidization.

The Mo-rich carbides in the 1050-ST sample were completely dissolved (Figure 8(b2)). After holding at 860 °C for 4 h (the 1050-A sample, Figure 8(c2)), the re-precipitated Mo-rich carbides exhibited spherical, elongated, and irregular morphologies, with sizes mainly distributed in the range of 0.2–0.8 μm. Upon slow cooling from 860 °C to 740 °C (the 1050-B sample, Figure 8(d2)), both the number density and area fraction increased slightly, and the size was mainly distributed in the range of 0.2–0.8 μm. After holding at 740 °C for 8 h (the 1050-C sample, Figure 8(e2)), both the number density and area fraction increased substantially, with the size mainly distributed in the range of 0.2–1.0 μm. In the final spheroidized condition (the 1050-F sample, Figure 8(f2)), the number density of Mo-rich carbides was comparable to that at Point C, while the area fraction increased slightly, and the size was mainly distributed in the range of 0.1–1.2 μm. For the 1050-ST sample, the average size of Mo-rich carbides increased from 0.45 μm at Point A to 0.50 μm in the 1050-F sample, and the *d*_max_/*d*_min_ ratio increased to approximately 2.2, indicating a reduction in particle roundness.

The Mo-rich carbides in the 1150-ST sample were also completely dissolved (Figure 8(b3)). After holding at 860 °C for 4 h (the 1150-A sample, Figure 8(c3)), no Mo-rich carbides precipitated. Not until the temperature was lowered to 740 °C (the 1150-B sample, Figure 8(d3)) did a small amount of Mo-rich carbides appear, exclusively along grain boundaries, exhibiting chain-like and irregular morphologies. After further holding at 740 °C for 8 h (the 1150-C sample, Figure 8(e3)), the Mo-rich carbides were predominantly distributed along grain boundaries in chain-like and irregular forms, while a very small number of spherical Mo-rich carbides (0.1–0.3 μm) were also observed within the grains. Both the number density and area fraction of Mo-rich carbides increased substantially, with the size mainly distributed in the range of 0.2–1.0 μm. In the final spheroidized condition (the 1150-F sample, Figure 8(f3)), the morphology of Mo-rich carbides was similar to that at Point C, being mainly distributed along grain boundaries in chain-like and irregular forms, with only a very small number of fine Mo-rich carbides (0–0.3 μm) present within the grains. The average size of Mo-rich carbides in the 1150-ST sample decreased from 0.79 μm at Point B to 0.54 μm in the final spheroidized condition, which is attributed to the precipitation of a small fraction of fine Mo-rich carbides within the grains. The d_max_/d_min_ ratio of Mo-rich carbides in the 1150-F sample reached 2.6, indicating poor roundness, and the particles along grain boundaries were interconnected, exhibiting irregular chain-like or network morphologies.

### 3.5. Effect of Solution Temperature on the V-Shaped Notch Impact Energy

The V-notch Charpy impact results are shown in Table 3 and Figure 10. The Rockwell hardness of all the quenched-and-tempered samples lies in a narrow range of 44–46 HRC, indicating that the samples possess comparable strength levels. With increasing solution temperature, the impact energy first increased and then decreased, reaching a maximum of approximately 18.3 J at 1000 °C. The impact energy of the 950-F and 1050-F samples was approximately 16.6 J and 17.6 J, respectively, while further increasing the solution temperature to 1100 °C and 1150 °C led to a pronounced decrease to approximately 14.8 J and 13.0 J, respectively. This variation correlates closely with the grain size and the distribution of the Mo-rich carbides.

The 950-F and 1000-F samples contained fine, uniformly dispersed Mo-rich carbides within the grains with no grain-boundary carbide network (Figure 5a,b), which corresponds to the relatively high impact energy. With increasing solution temperature, the Mo-rich carbides coarsened, their d_max_/d_min_ ratio increased, and they gradually segregated to the grain boundaries, forming chain-like and network morphologies (Figure 5c–f); simultaneously, the matrix grains coarsened. Such a spheroidized microstructure is unfavorable to impact toughness, consistent with the pronounced decrease in the impact energy of the 1100-F and 1150-F samples.

### 3.6. Morphological Characteristics and Precipitation Behavior of Mo-Rich Carbides

The preceding sections have analyzed the effect of different solution temperatures on the morphology and size distribution of Mo-rich carbides in the experimental steel. With increasing solution temperature, the Mo-rich carbides in the F-state samples exhibited a reduced quantity, increased size, decreased roundness, and a tendency toward grain boundary segregation. To further understand the morphological characteristics and precipitation behavior of Mo-rich carbides, this section analyzes their precipitation morphology features.

Figure 11 presents secondary electron images showing the morphology details of Mo-rich carbides in the 950-F, 1050-F, and 1150-F samples. In the 950-F sample, Mo-rich carbides were predominantly distributed within the grains in a spherical morphology (Figure 11a). In the 1050-F sample, in addition to intragranular spherical carbides, chain-like distributions were also observed along grain boundaries (Figure 11b). In the 1150-F sample, Mo-rich carbides were mainly distributed along grain boundaries in chain-like and network morphologies (Figure 11c).

Based on the foregoing analysis, at relatively low solution temperatures (950 °C, 1000 °C), Mo-rich, Cr-rich, and V-rich carbides all partially remained after solution treatment. The Mo-rich carbides of samples after spheroidizing annealing could be classified into two categories: one part consisted of the residual Mo-rich carbides inherited from the solution treatment, and the other part comprised newly precipitated Mo-rich carbides nucleating on pre-existing Cr-rich carbides, V-rich carbides, and grain boundaries. In contrast, at higher solution temperatures (1050–1150 °C), both Mo-rich and Cr-rich carbides were completely dissolved after solution treatment, leaving only some V-rich carbides. Under these conditions, Mo-rich carbides could only nucleate on residual V-rich carbides and grain boundaries. Notably, in the 1150-A sample (Figure 8(c3)), a large number of fine Cr-rich carbides precipitated, whereas no Mo-rich carbides were detected, indicating that Cr-rich carbides precipitate more readily and earlier than Mo-rich carbides. It should be noted that the pre-precipitated Cr-rich carbides during spheroidizing annealing may also serve as nucleation sites for subsequent Mo-rich carbide precipitation.

To verify the above possibility, the 950-F and 1050-F samples were examined under TEM. Figure 12 presents the STEM images and corresponding EDS mapping images of the 950-F sample. It can be observed that some Mo-rich carbides existed independently within the grains, while others were adjacent to Cr-rich and V-rich carbides. However, this observation alone cannot conclusively prove that Mo-rich carbides nucleate on Cr-rich or V-rich carbides, as the Mo-rich carbides observed here could also be those retained from the low-temperature solution treatment. Therefore, it is necessary to examine the 1050-F sample.

Figure 13 presents TEM images of the 1050-F sample. In Figure 13a, a fine V-rich carbide is embedded at the center of a Mo-rich carbide, indicating that the Mo-rich carbide nucleated on the V-rich carbide as a nucleation site. Figure 13b shows Mo-rich, Cr-rich, and V-rich carbides adjacent to one another within the grain, suggesting that Cr-rich and Mo-rich carbides precipitated successively in the vicinity of V-rich carbides. Figure 13c,d display Mo-rich carbides precipitated adjacent to Cr-rich carbides. Previous studies have reported that Mo-rich M_2_C and M_6_C carbides can nucleate on Cr-rich M_7_C_3_ and M_23_C_6_ carbides. This is because, during the nucleation of M_7_C_3_ and its subsequent transformation to M_23_C_6_, Mo atoms diffuse from M_7_C_3_ and the matrix to the interface between M_7_C_3_ and the matrix, where they accumulate to form M_2_C or M_6_C [5,24]. Figure 13e,f show Mo-rich carbides precipitated at grain boundaries, exhibiting irregular morphologies. It should be noted that what is demonstrated experimentally here is the spatial association between the Mo-rich carbides and the pre-existing Cr-rich and V-rich carbides (Figure 12 and Figure 13). The Mo-rich carbides nucleated on these carbides is an interpretation of this observation, supported by previous studies [5,24].

### 3.7. Mechanism of Mo-Rich Carbide Evolution

This subsection elucidates the evolution mechanism of Mo-rich carbides in the experimental steel during solution treatment and spheroidizing annealing from the perspectives of thermodynamic conditions, atomic diffusion and nucleation sites, while clarifying the regulatory role of different solution temperatures on the evolution of Mo-rich carbides.

(1) Thermodynamic conditions: Temperature dependence of Mo solubility and Mo-rich carbide precipitation

The solubility of Mo atoms in the experimental steel matrix increases with rising temperature [5]. During the solution treatment stage, a higher temperature leads to greater Mo solubility, thereby resulting in a higher degree of dissolution of Mo-rich carbides. At solution temperatures of 950 °C and 1000 °C, the solubility of Mo atoms is relatively low; consequently, only a portion of the Mo-rich carbides dissolves into the matrix. When the solution temperature reaches 1050 °C or above, the solubility of Mo atoms increases substantially, and the Mo-rich carbides are completely dissolved.

During the spheroidizing annealing stage, as the temperature decreases from 860 °C to a final slow cooling to 500 °C, the solubility of Mo atoms continuously decreases. Supersaturated Mo atoms combine with C atoms to form and precipitate Mo-rich carbides, which provides the thermodynamic driving force for the precipitation of Mo-rich carbides. The isothermal holding steps and slow cooling provide sufficient time for the diffusion of Mo atoms and the spheroidization of carbides, enabling the carbides to undergo Ostwald ripening [26].

(2) Atomic diffusion: Restriction of Mo diffusion characteristics on the nucleation sites of Mo-rich carbides

As shown in Figure 14, the diffusion coefficient of Mo atoms in steel is considerably lower than that of alloying elements such as Cr and V, both at solution temperatures and within the spheroidizing annealing temperature range [27]. This characteristic results in significant differences in the precipitation and growth behavior of Mo-rich carbides compared with Cr-rich and V-rich carbides. Because the diffusion coefficient of Mo is substantially lower than that of Cr in the solution temperature range, after complete dissolution of Mo-rich carbides at solution temperatures of 1050–1150 °C, the diffusion of Mo becomes difficult. Furthermore, within the spheroidizing annealing temperature range, the diffusion coefficient of Cr-rich carbides remains much higher than that of Mo-rich carbides, making it easier for Cr-rich carbides to nucleate spontaneously and precipitate. In contrast, Mo-rich carbides require nucleation sites such as residual carbides or grain boundaries for precipitation. Therefore, with increasing solution temperature, the total number density of carbides after spheroidizing annealing increases, whereas the number density of Mo-rich carbides decreases (Figure 7c,d, Supplementary Material Figure S1).

(3) Nucleation sites: Regulation of Mo-rich carbide nucleation modes by solution temperature

The type and number of nucleation sites are key factors determining the precipitation and distribution of Mo-rich carbides. The nucleation of Mo-rich carbides can be categorized into two modes: nucleation on carbides and nucleation at grain boundaries. The solution temperature determines the nucleation mode of Mo-rich carbides by modifying the residual state of carbides prior to spheroidizing annealing, as illustrated in Figure 15.

Low-temperature solution treatment (950 °C, 1000 °C): After solution treatment, the grains are fine. A small number of fine carbides remain in the matrix, providing abundant nucleation sites (Cr-rich and V-rich carbides) for the precipitation of Mo-rich carbides during spheroidizing annealing. Consequently, Mo atoms, characterized by low diffusivity, can precipitate uniformly and dispersedly within the grains, forming numerous fine, near-spherical granular Mo-rich carbides. Notably, at a solution temperature of 1000 °C, although the Mo-rich carbides are largely dissolved, the remaining carbides provide an appropriate number of exogenous nucleation sites (Cr-rich and V-rich carbides) for Mo-rich carbides. This results in a slightly higher number density of Mo-rich carbides after spheroidizing annealing compared with the 950-ST sample, indicating that 1000 °C provides the most favourable combination of the investigated microstructural characteristics among the conditions examined, balancing the carbide number density after spheroidizing annealing with the uniform and dispersed precipitation of Mo-rich carbides.

High-temperature solution treatment (1050 °C, 1100 °C, 1150 °C): After solution treatment, the grains are coarser. Only a very small number of V-rich carbides remain in the matrix. The nucleation sites for Mo-rich carbides within the grains are severely lacking, and supersaturated Mo atoms predominantly aggregate at grain boundaries to form Mo-rich carbides. Grain boundaries possess higher energy and are thus preferred nucleation sites [28]. Moreover, grain boundaries serve as fast diffusion channels for atomic transport, causing Mo atoms to continuously migrate toward and accumulate at grain boundaries. This leads to the nucleation, growth, and eventual interconnection of Mo-rich carbides at grain boundaries, ultimately forming network carbides distributed along the grain boundaries.

In summary, the evolution of Mo-rich carbides during post-forging solution treatment and spheroidizing annealing is the synergistic result of thermodynamic conditions, atomic diffusion, and nucleation sites. The solution temperature determines the solubility of Mo atoms and the distribution of nucleation sites. The low diffusivity of Mo atoms dictates that their nucleation during spheroidizing annealing relies on residual carbides and grain boundaries. The isothermal holding steps and slow cooling process during spheroidizing annealing provide both the thermodynamic driving force and sufficient time for the precipitation and growth of Mo-rich carbides.

## 4. Conclusions

In this study, the evolution of Mo-rich carbides in H13 hot-working die steel during spheroidizing annealing at different solution temperatures ranging from 950 °C to 1150 °C was systematically investigated by means of JMatPro thermodynamic calculations combined with OM, SEM, TEM, and XRD characterization techniques, together with V-notch Charpy impact and Rockwell hardness tests. The regulatory effect of solution temperature on the morphology, size, and distribution of Mo-rich carbides was clarified. Furthermore, the evolution mechanism of Mo-rich carbides was elucidated from three aspects: thermodynamics, atomic diffusion, and nucleation sites. The main conclusions are as follows:

(1) The difficulty of carbide dissolution follows the order: V-rich carbides (MC) > Mo-rich carbides (M_2_C/M_6_C) ≈ Cr-rich carbides (M_23_C_6_/M_7_C_3_). After low-temperature solution treatment (950–1000 °C), Mo-rich, Cr-rich, and V-rich carbides all partially remain. After high-temperature solution treatment (1050–1150 °C), only V-rich carbides remain, while all other carbides are completely dissolved.

(2) The solution temperature exerts a significant influence on the morphology and distribution of Mo-rich carbides in the experimental steel after spheroidizing annealing. At low solution temperatures (950–1000 °C), Mo-rich carbides predominantly precipitate within grains by nucleating on residual carbides (Cr-rich and V-rich carbides) after spheroidizing annealing, appearing as uniformly dispersed near-spherical particles without grain boundary segregation. At solution temperatures of 1050–1150 °C, the Mo-rich carbides in the spheroidizing-annealed samples gradually segregate toward grain boundaries as the solution temperature increases. With increasing solution temperature, the number of Mo-rich carbides in the spheroidizing-annealed samples decreases, while their average size and *d*_max_/*d*_min_ ratio increase, accompanied by an increasing degree of irregularity.

(3) The solution temperature determines the solubility of Mo atoms and the distribution of nucleation sites. The low diffusivity of Mo atoms dictates that their nucleation during spheroidizing annealing relies on residual carbides and grain boundaries. The isothermal holding steps and slow cooling process during spheroidizing annealing provide both the thermodynamic driving force and sufficient time for the precipitation and growth of Mo-rich carbides.

(4) Considering the morphology, size, distribution, and spheroidization quality of Mo-rich carbides comprehensively, 1000 °C provides the most favourable combination of the investigated microstructural characteristics among the conditions examined for the experimental hot-working die steel after forging. This temperature achieves a balance between the carbide number density after spheroidizing annealing and the uniform dispersed precipitation of Mo-rich carbides. After quenching and triple tempering, the Rockwell hardness of all the samples lies within a narrow range of 44–46 HRC, indicating comparable strength levels. The V-notch impact energy reaches its highest value of 18.3 J at this temperature (compared with 13.0 J at 1150 °C). The optimum at 1000 °C thereby provides a favorable microstructural foundation for the subsequent quenching and tempering process of H13 steel.

## Figures and Tables

**Figure 1:**
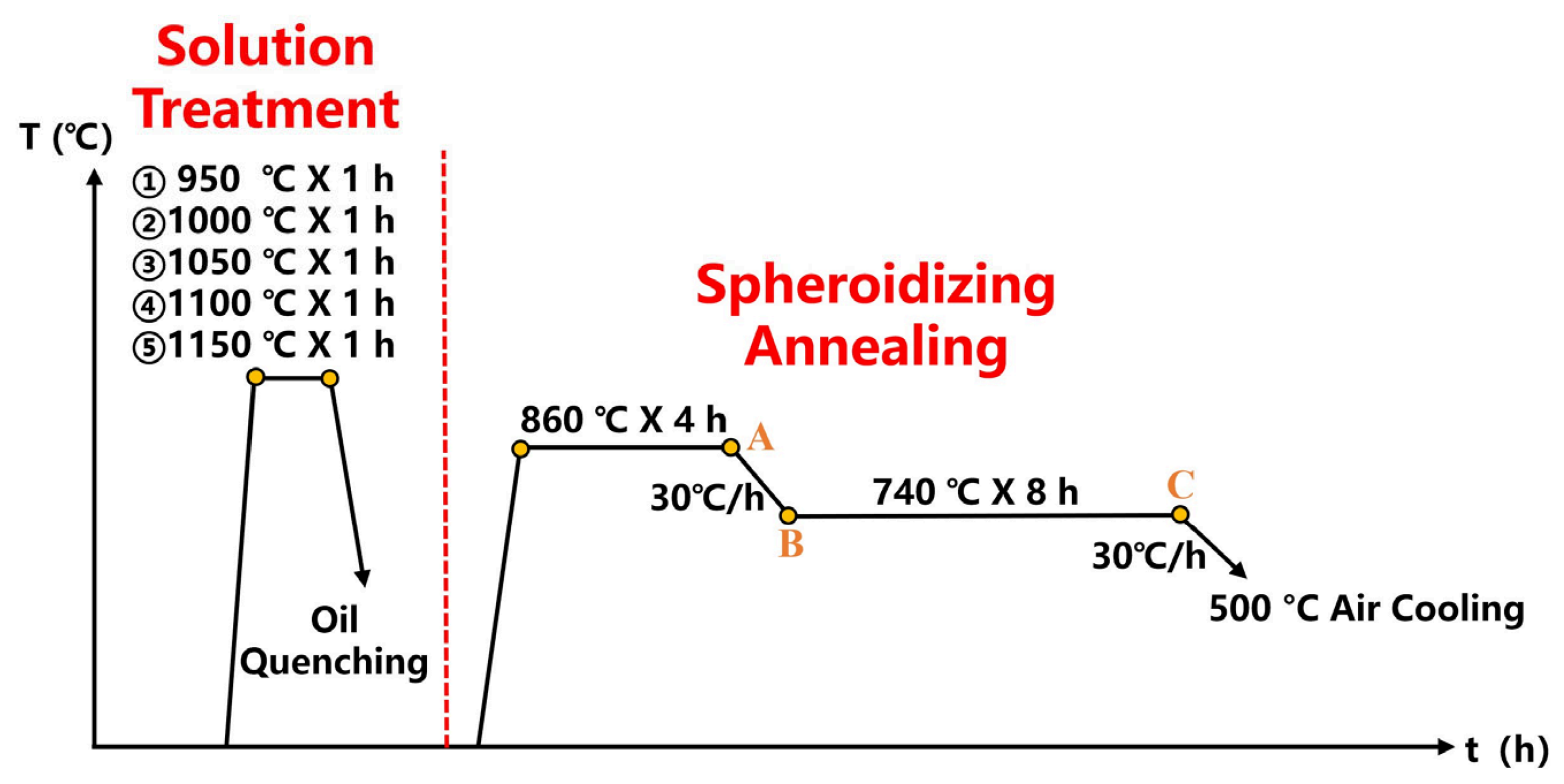
Heat treatment process. The red dotted line separates the solution treatment stage from the spheroidizing annealing stage.

**Figure 2:**
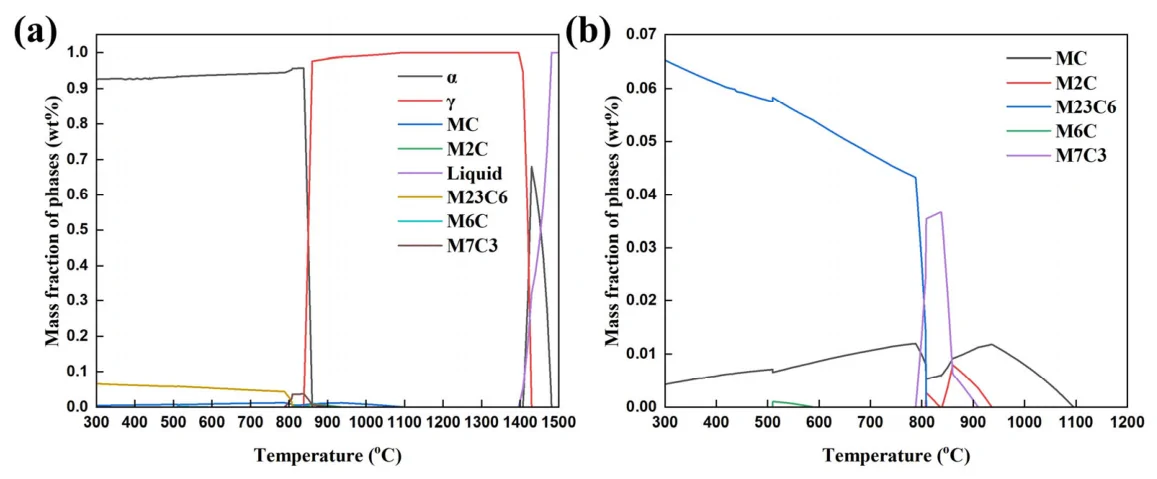
Equilibrium carbide diagram of the experimental steel: (**a**) mass fractions of the phases over the whole temperature range; (**b**) magnified view of the carbide precipitation range (300–1200 °C).

**Figure 3:**
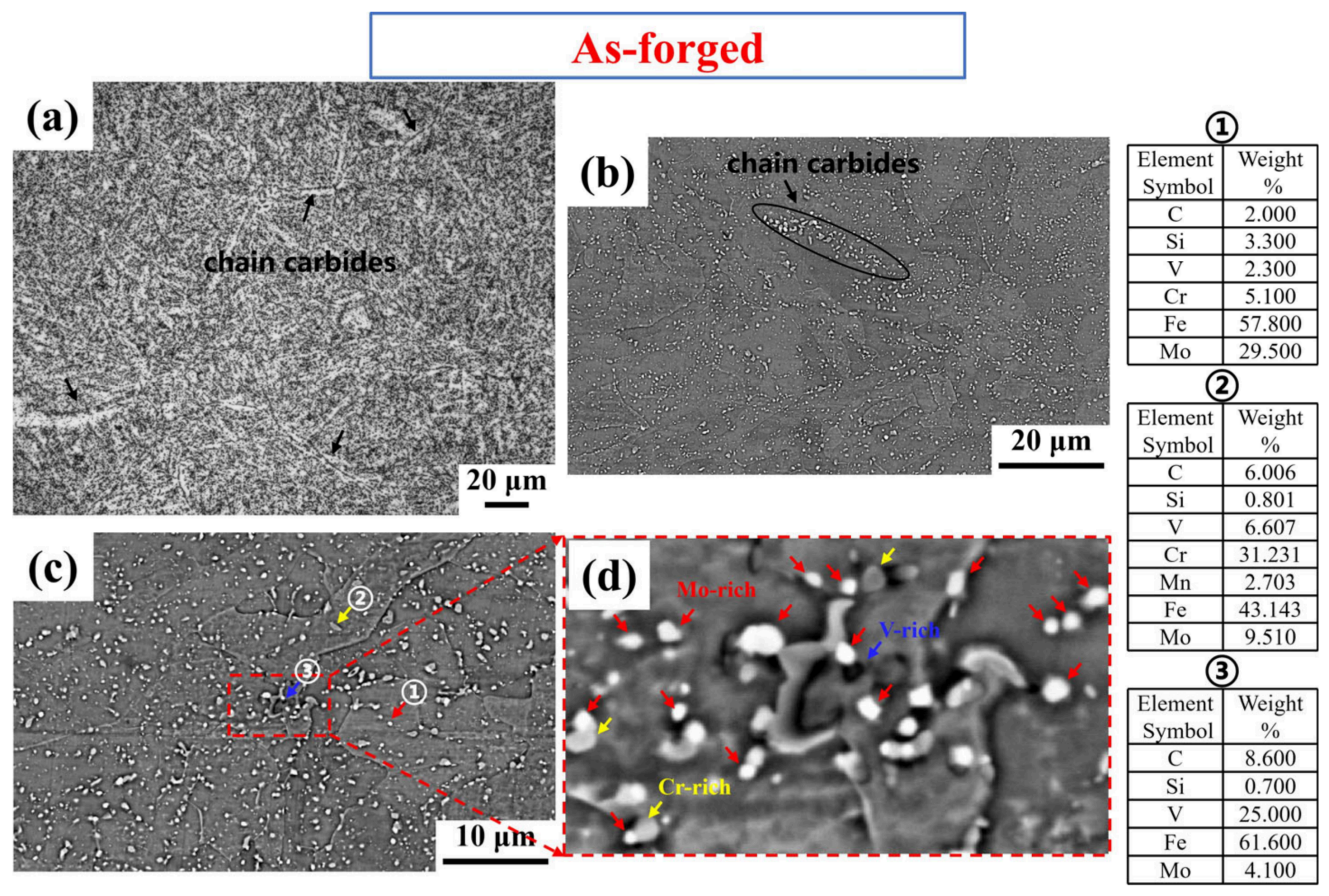
Microstructure of as-forged and softening-annealed samples. (**a**) Optical micrograph. (**b**,**c**) Backscattered electron images. (**d**) Magnified view of the region outlined by the dashed red rectangle in (**c**). Chain carbides are indicated by black arrows in (**a**,**b**). Mo-rich, Cr-rich, and V-rich carbides are marked by red, yellow, and blue arrows, respectively, in (**c**,**d**).

**Figure 4:**
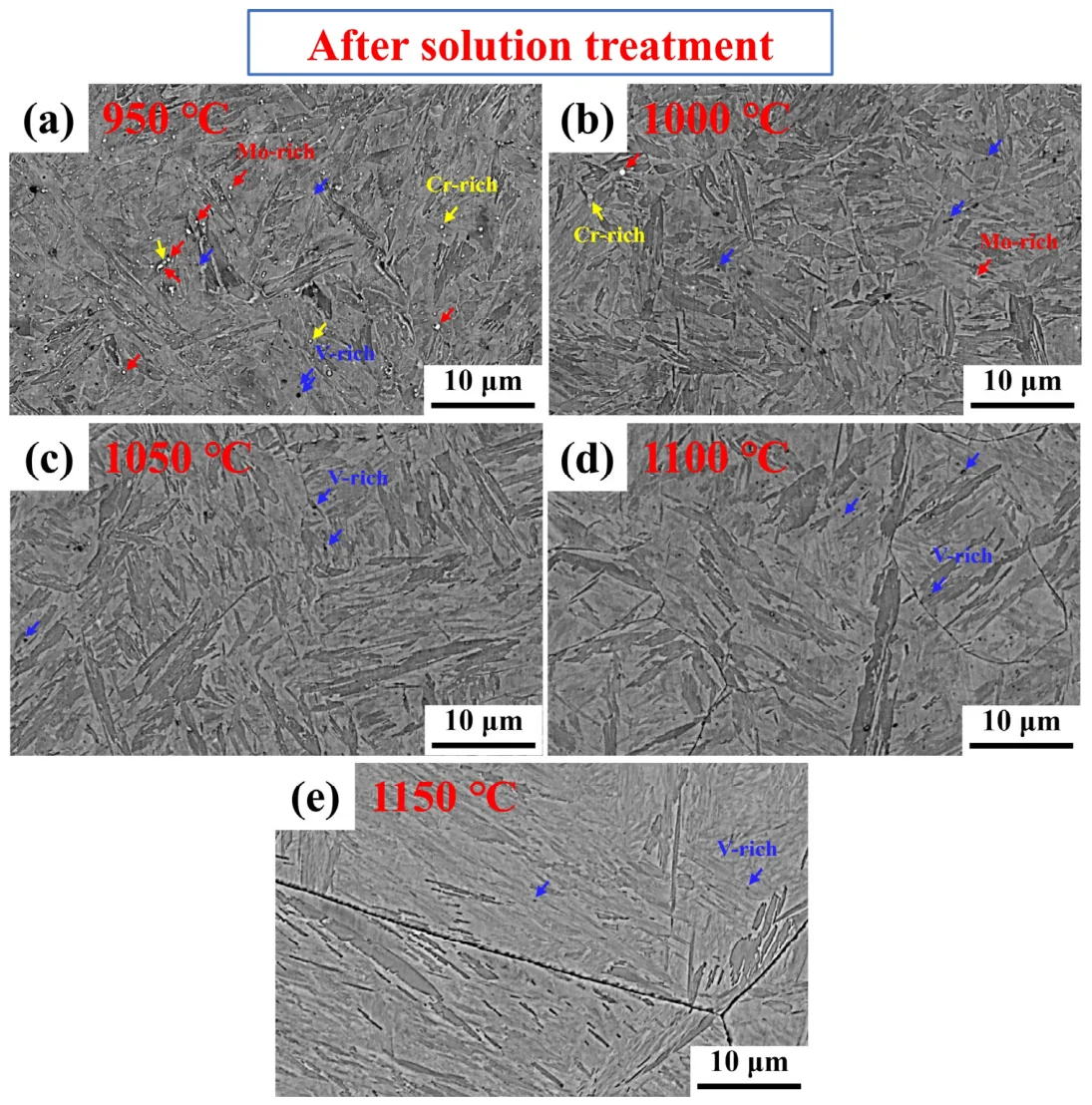
Backscattered electron images of experimental steel after solid solution treatment at different temperatures. (**a**) 950-ST; (**b**) 1000-ST; (**c**) 1050-ST; (**d**) 1100-ST; (**e**) 1150-ST. Mo-rich, Cr-rich, and V-rich carbides are marked by red, yellow, and blue arrows, respectively.

**Figure 5:**
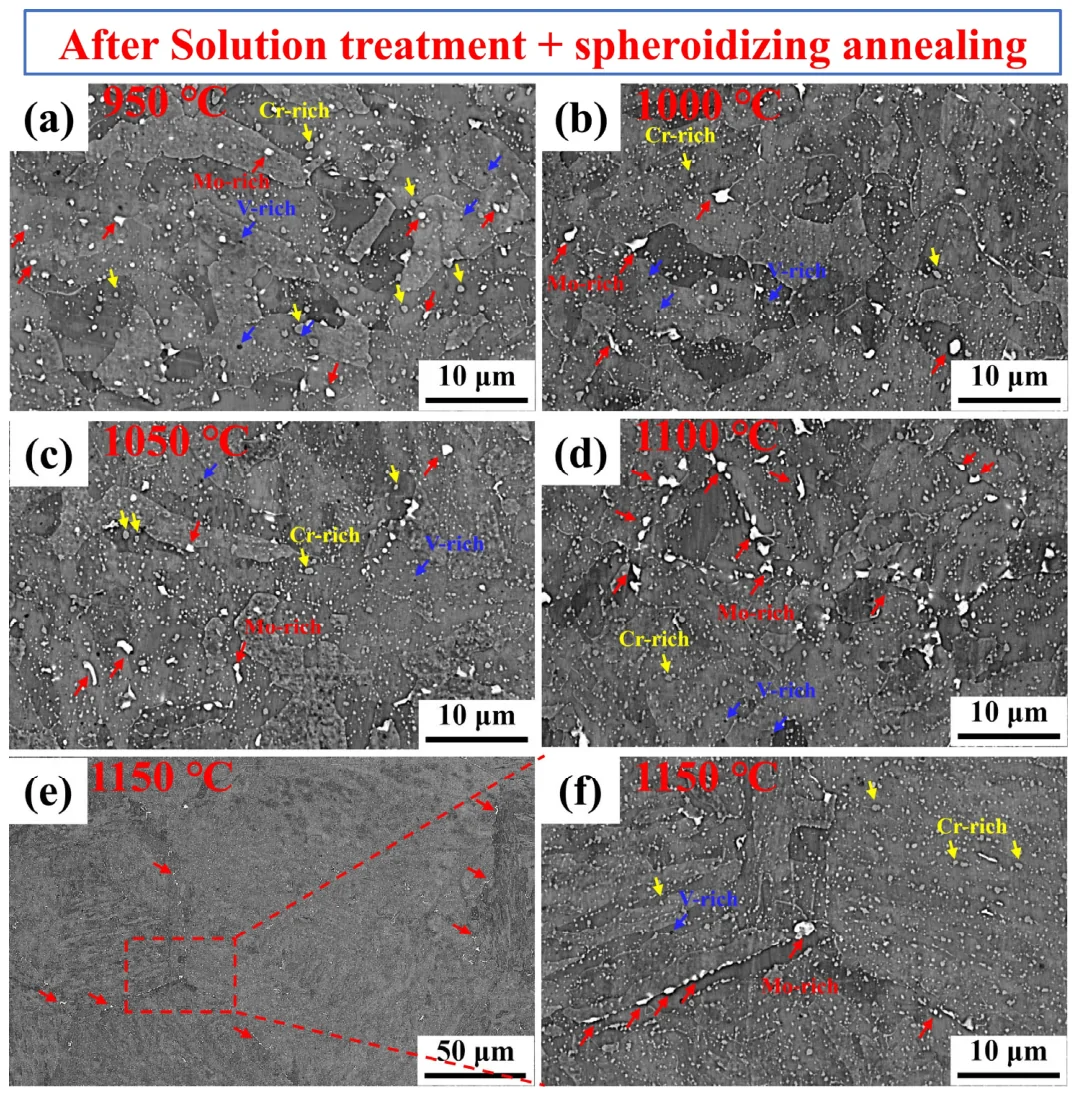
Backscattered electron images of the experimental steel after solution treatment at different temperatures followed by spheroidizing annealing. (**a**) 950-F; (**b**) 1000-F; (**c**) 1050-F; (**d**) 1100-F; (**e**) 1150-F; (**f**) magnified view of the region outlined by the red dashed rectangle in (**e**); Mo-rich, Cr-rich, and V-rich carbides are marked by red, yellow, and blue arrows, respectively.

**Figure 6:**
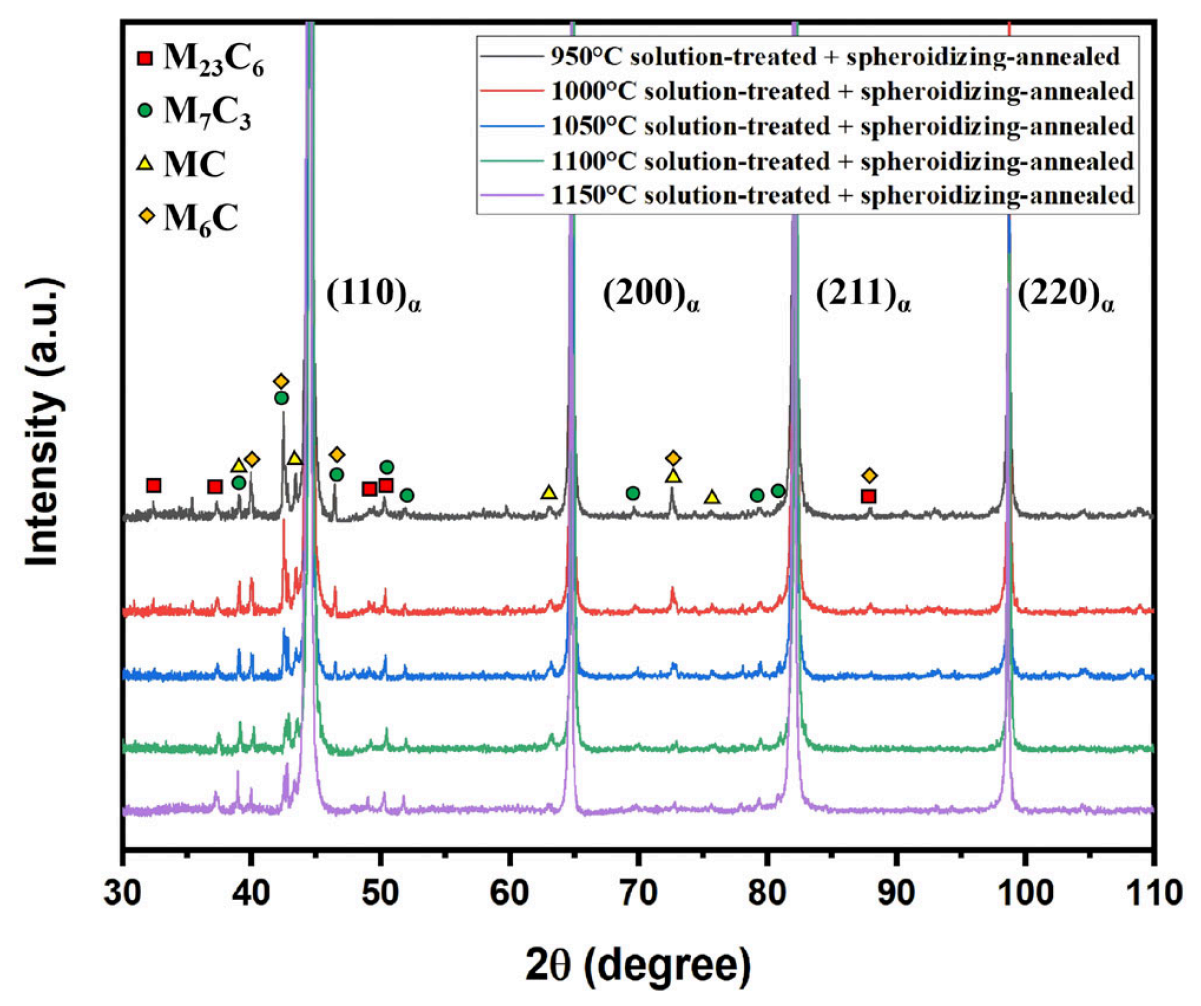
XRD patterns of the experimental steel after solution treatment at different temperatures followed by spheroidizing annealing.

**Figure 7:**
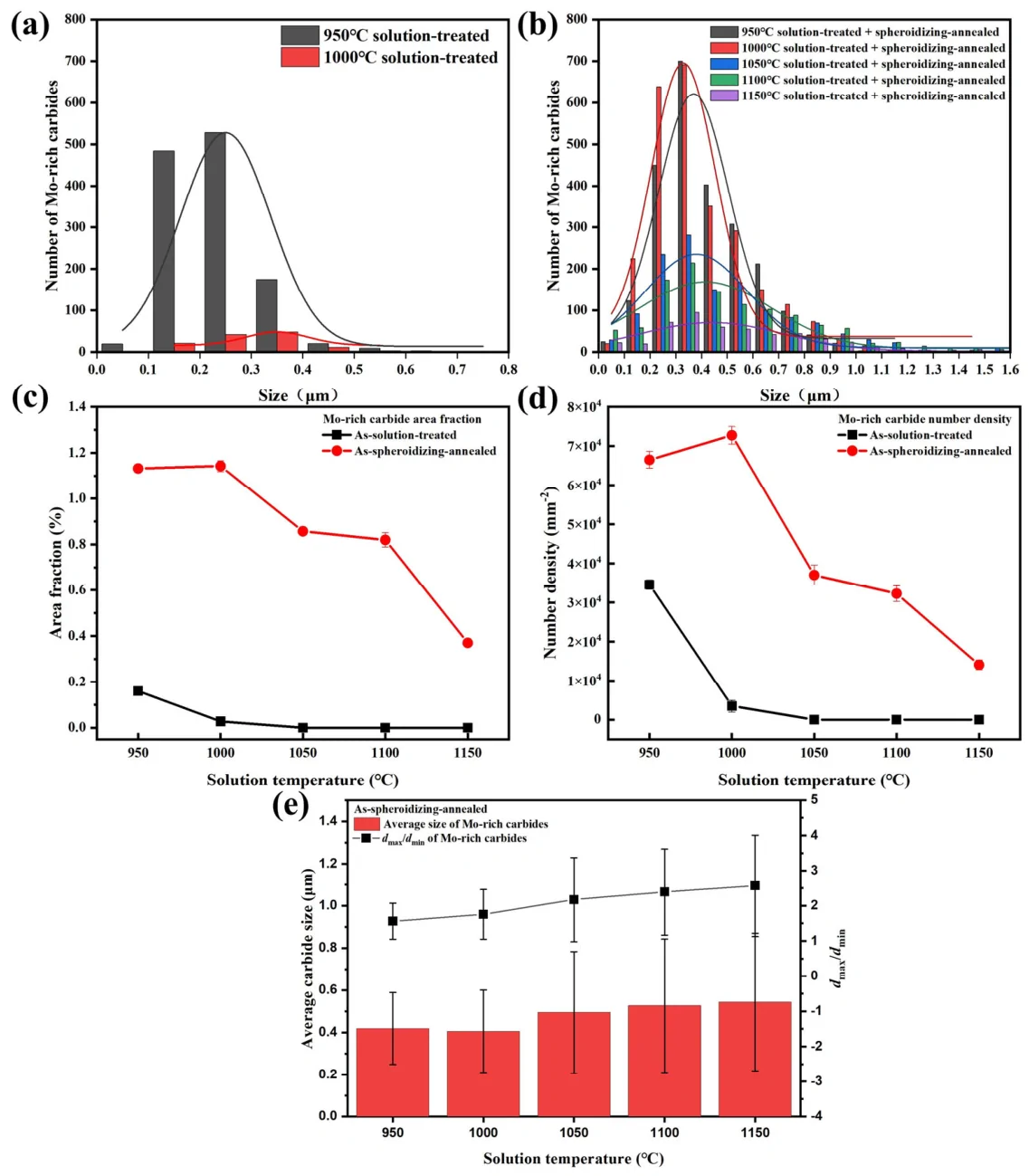
Size distribution of Mo-rich carbides in the experimental steel at different solution temperatures: (**a**) ST and (**b**) F conditions. Size statistics of Mo-rich carbides in the experimental steel at different solution temperatures. (**c**) Area fraction evolution, (**d**) number density evolution; (**e**) average size and d_max_/d_min_ ratio of Mo-rich carbides.

**Figure 8:**
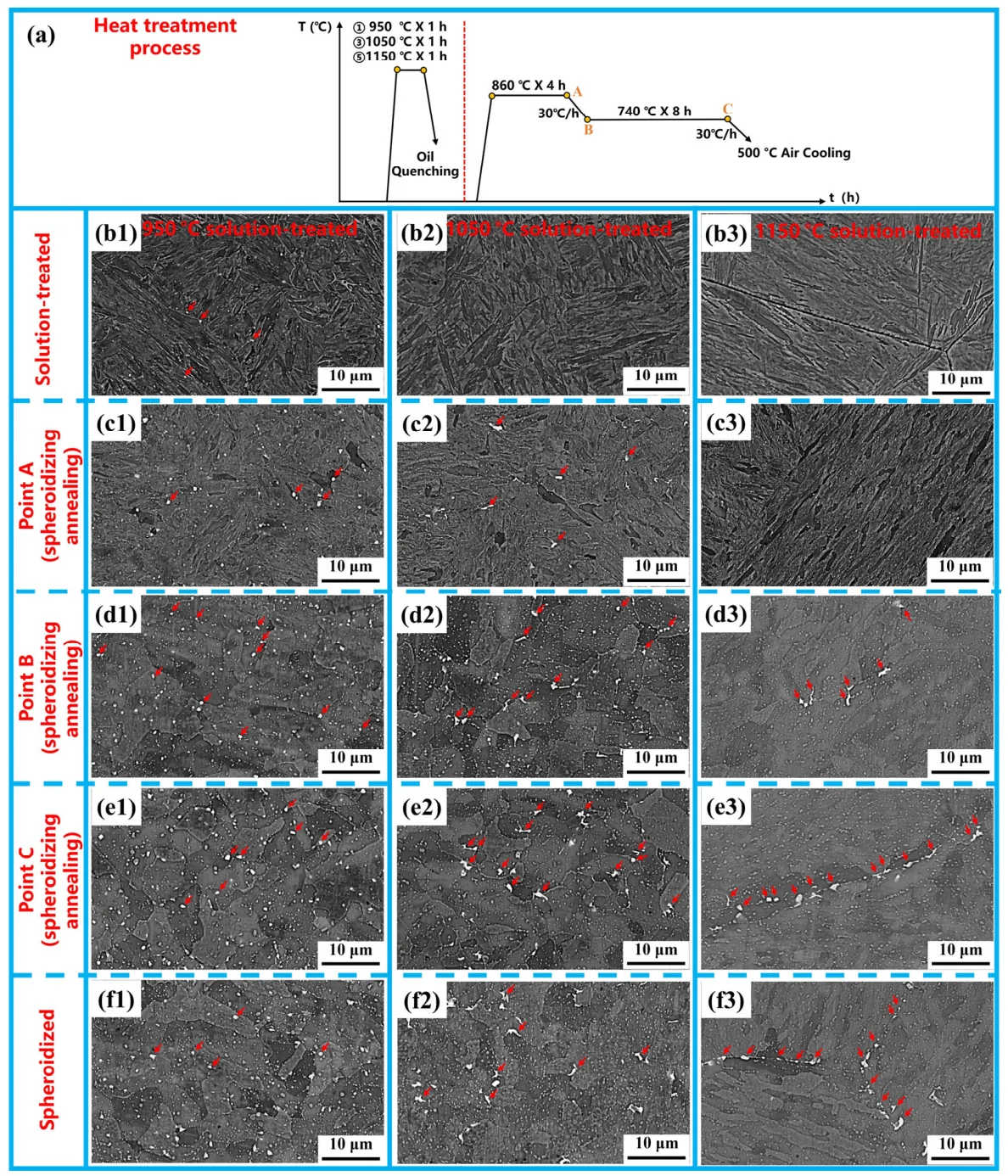
Morphological changes in Mo-rich carbides during heat treatment of the experimental steel at different solid solution temperatures. (**a**) Heat treatment process; (**b1**–**b3**) 950-ST, 1050-ST, and 1150-ST samples; (**c1**–**c3**) 950-A, 1050-A, and 1150-A samples; (**d1**–**d3**) 950-B, 1050-B, and 1150-B samples; (**e1**–**e3**) 950-C, 1050-C, and 1150-C samples; (**f1**–**f3**) 950-F, 1050-F, and 1150-F samples; Mo-rich carbides are marked by red arrows.

**Figure 9:**
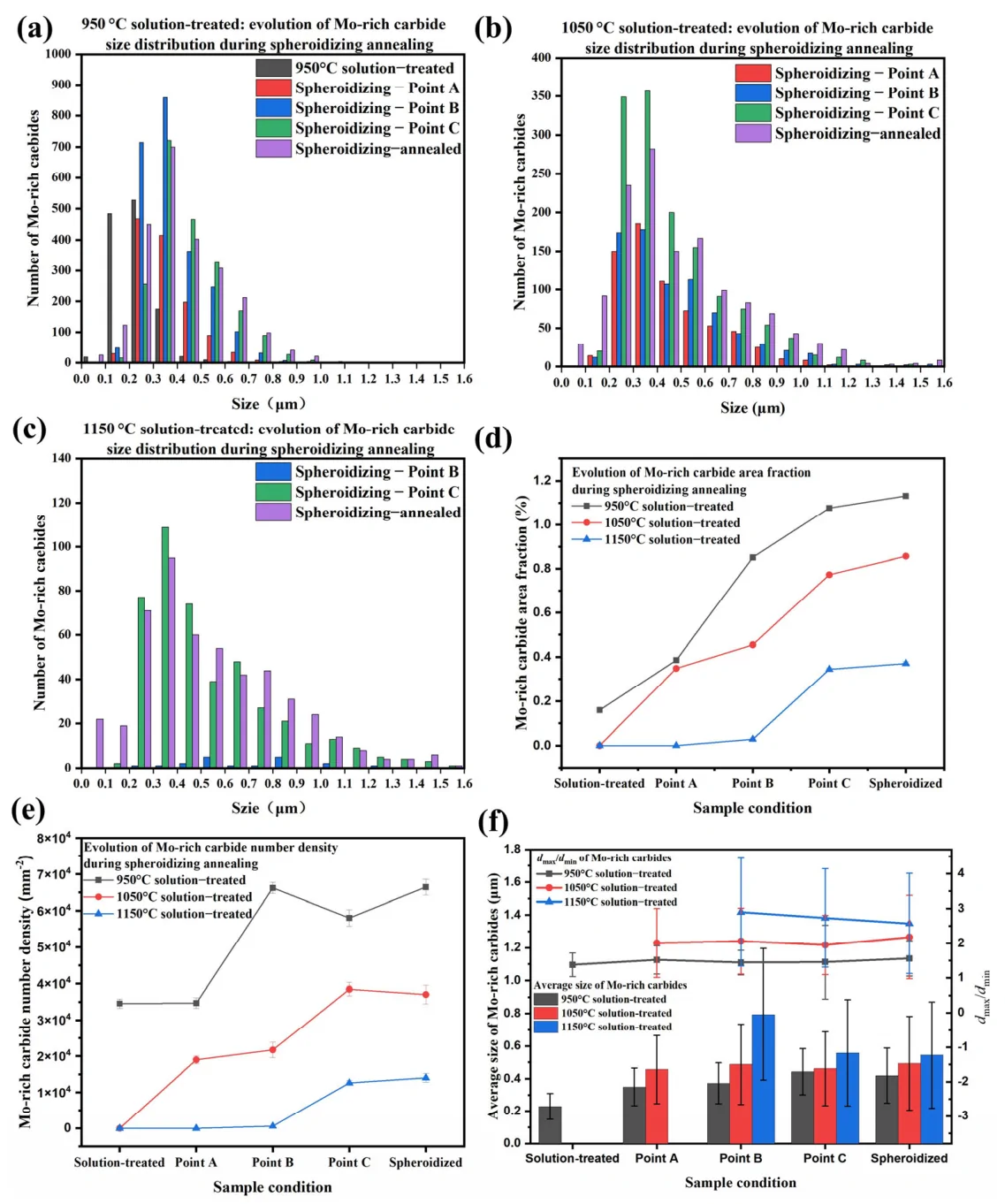
Size distribution and statistics of Mo-rich carbides during spheroidizing annealing after solution treatment at 950 °C, 1050 °C, and 1150 °C. Evolution of Mo-rich carbide size distribution: (**a**) 950-ST, (**b**) 1050-ST, and (**c**) 1150-ST samples. (**d**) Area fraction evolution, (**e**) number density evolution, and (**f**) average size and d_max_/d_min_ ratio evolution of Mo-rich carbides.

**Figure 10:**
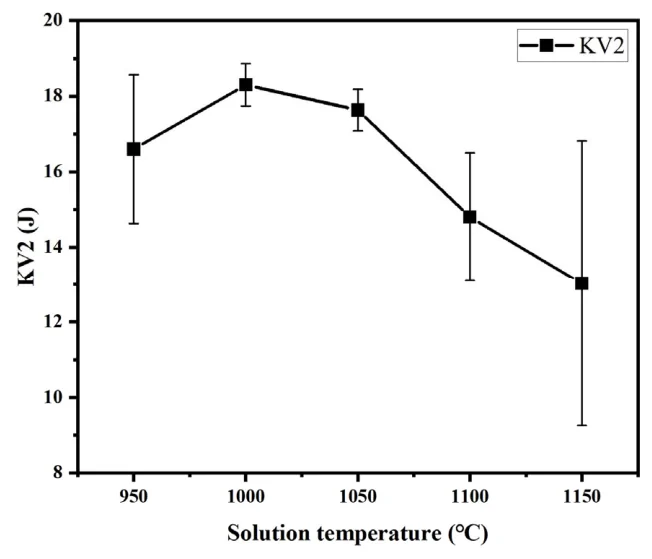
V-notch Charpy impact energy (KV2) of the experimental steel after solution treatment at different temperatures, spheroidizing annealing, and subsequent quenching and triple tempering. The error bars represent the standard deviation.

**Figure 11:**
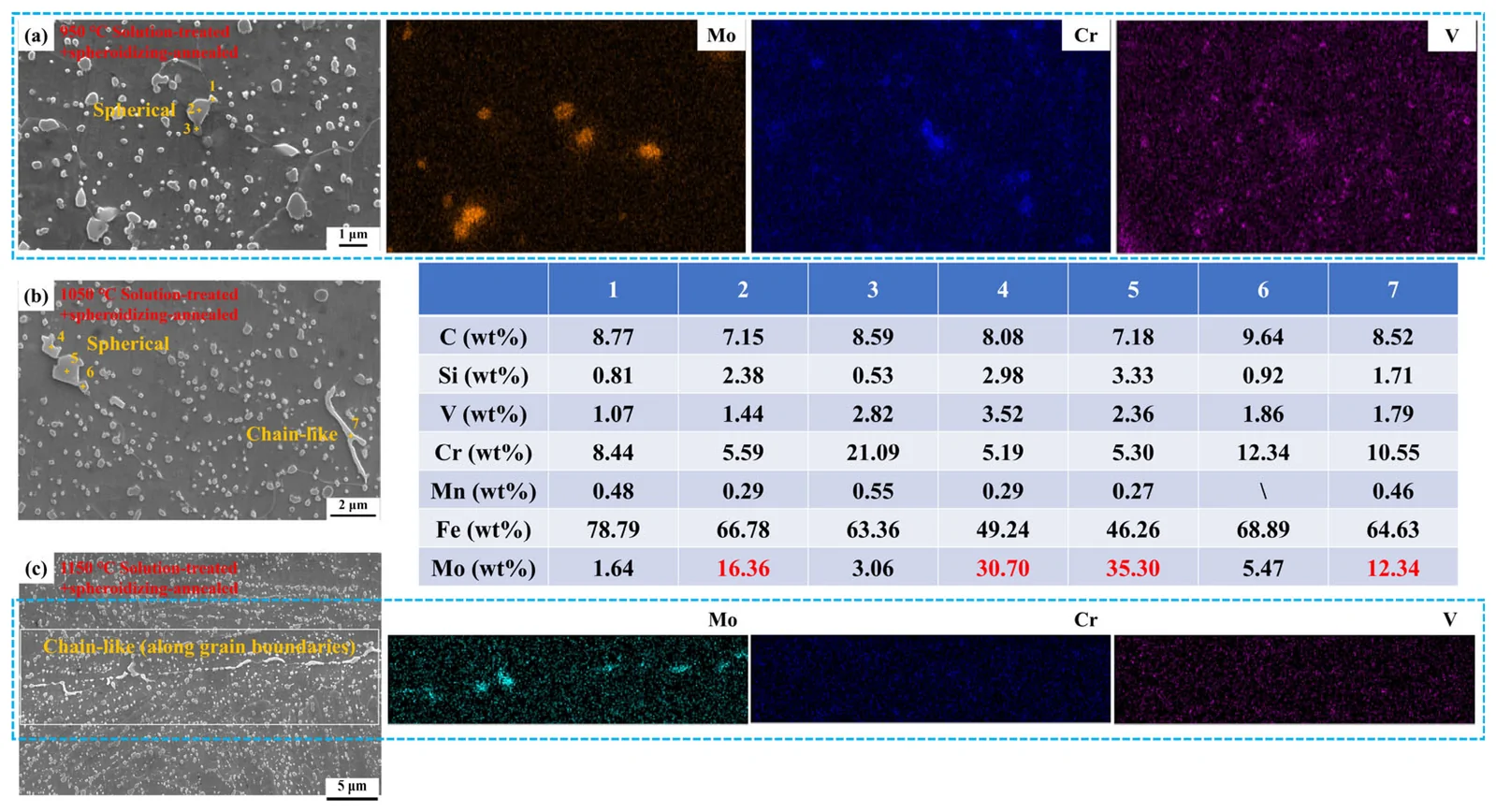
Secondary electron images of Mo-rich carbides in the experimental steel after spheroidizing annealing at different solution temperatures. (**a**) 950-F sample; (**b**) 1050-F sample; (**c**) 1150-F sample. The red text in each image indicates the heat-treatment condition of the sample, and the yellow text marks the distribution morphology of the Mo-rich carbides. The numbered points in (**a**,**b**) correspond to the EDS point-analysis results listed in the table in (**b**), and the Mo, Cr, and V elemental maps adjacent to each image show the corresponding elemental distributions.

**Figure 12:**
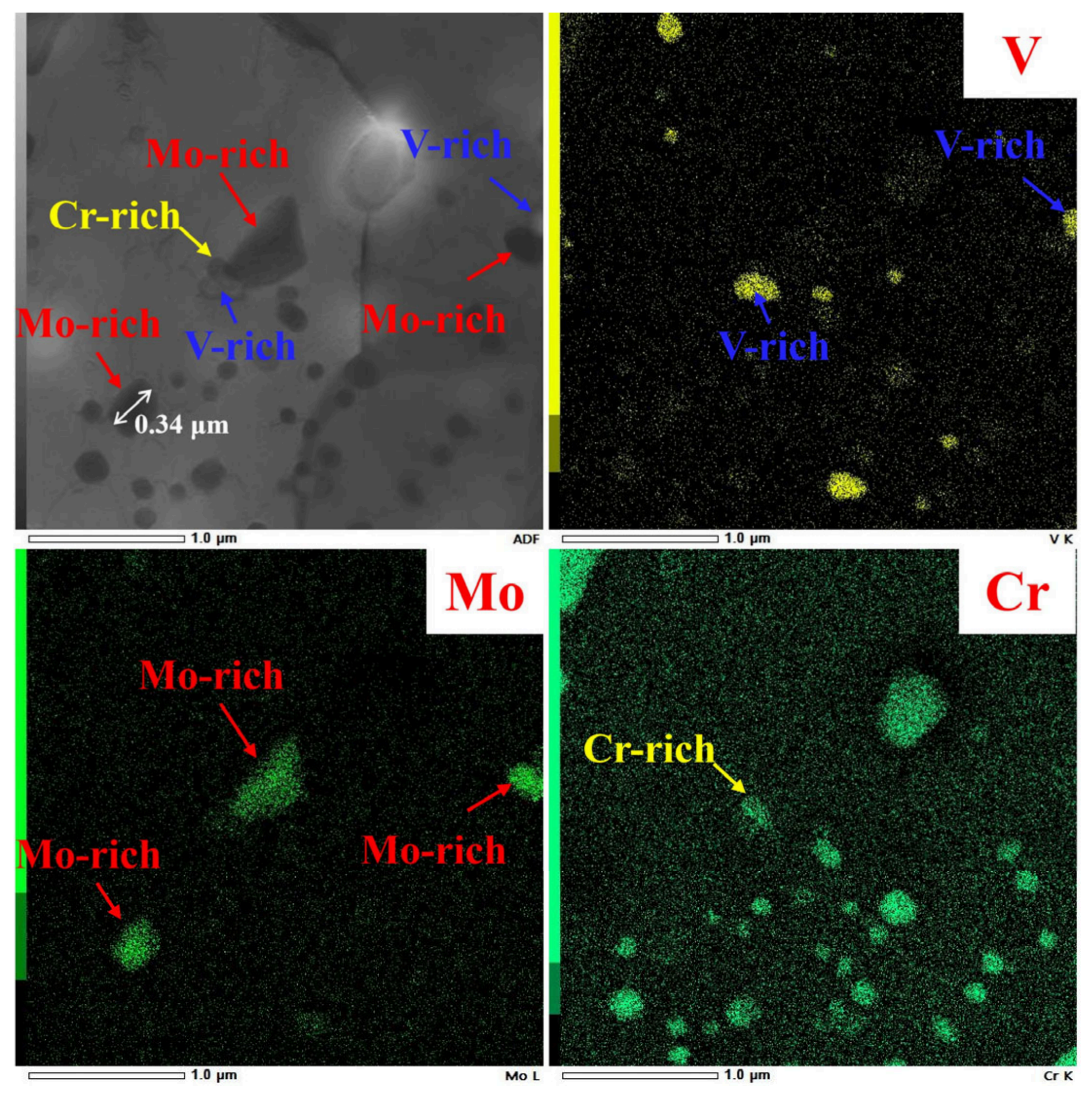
STEM images and EDS elemental mapping of the 950-F sample. Mo-rich, Cr-rich, and V-rich carbides are marked by red, yellow, and blue arrows, respectively.

**Figure 13:**
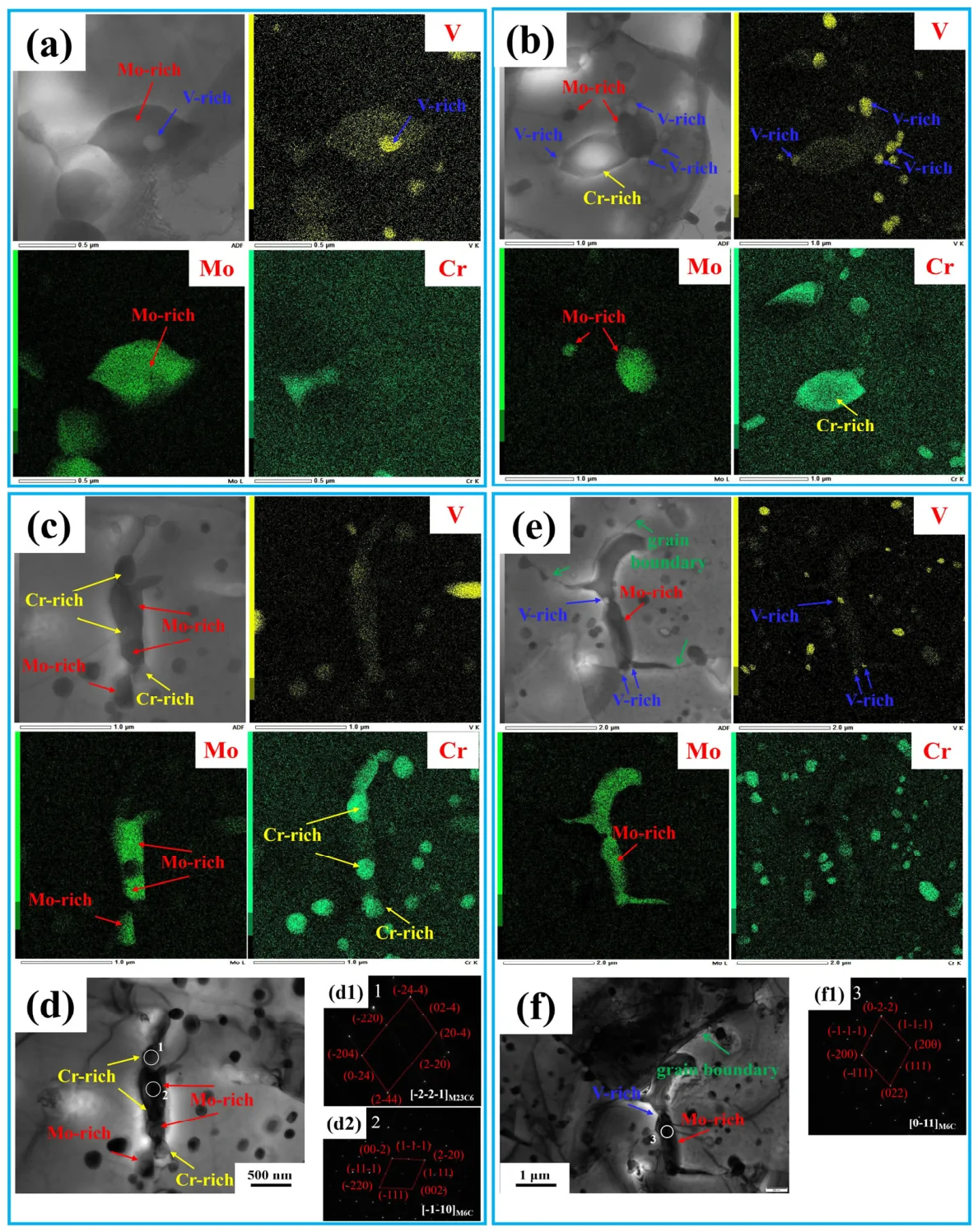
TEM images and EDS elemental mapping of the 1050-F sample. (**a**–**c**,**e**) STEM images and EDS elemental maps; (**d**,**f**) Bright-field TEM images; (**d1**,**d2**,**f1**) Selected-area electron diffraction (SAED) patterns. Mo-rich, Cr-rich, V-rich carbides and grain boundaries are marked by red, yellow, blue and green arrows respectively.

**Figure 14:**
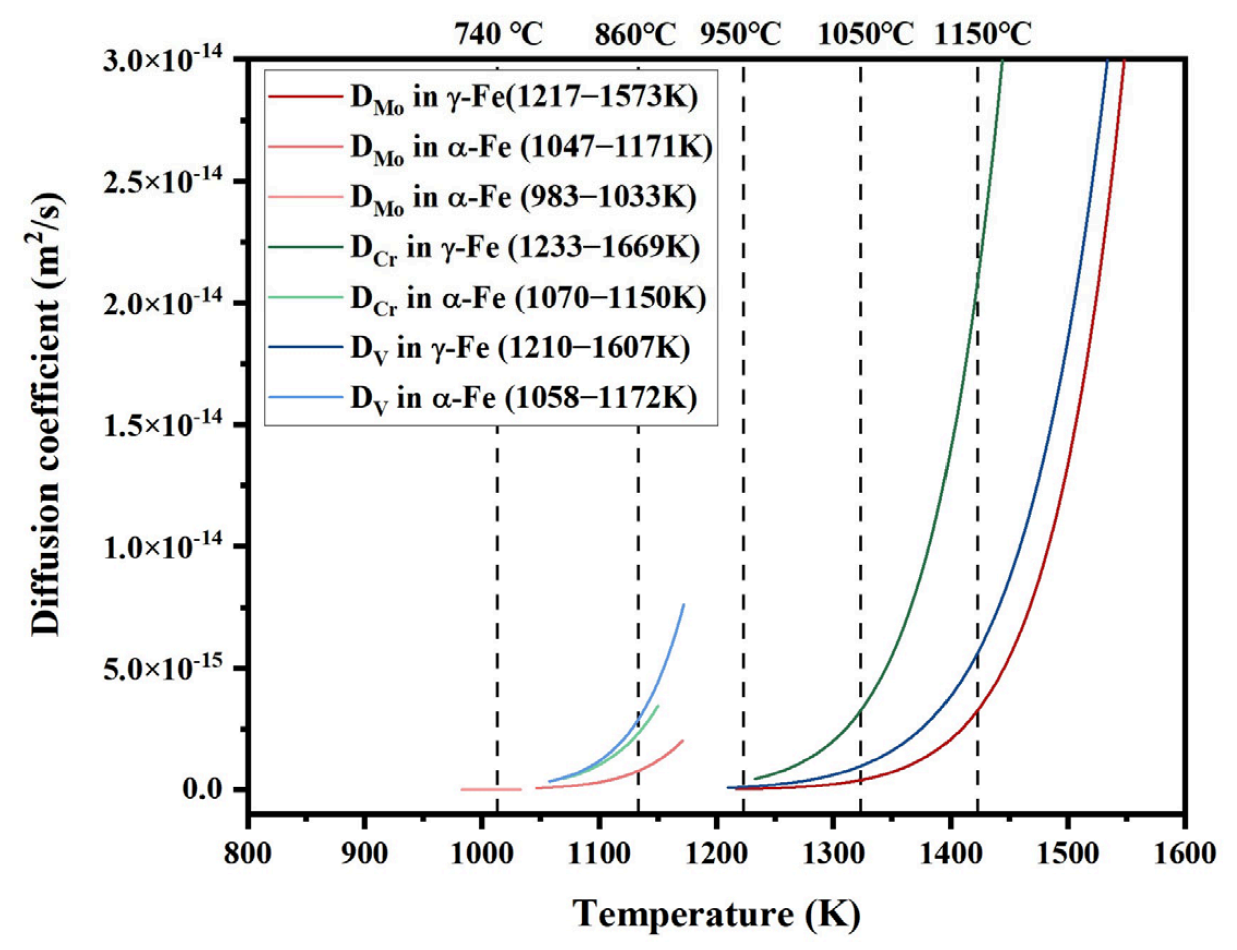
Variation in diffusion coefficients of Cr, Mo, and V in iron with temperature.

**Figure 15:**
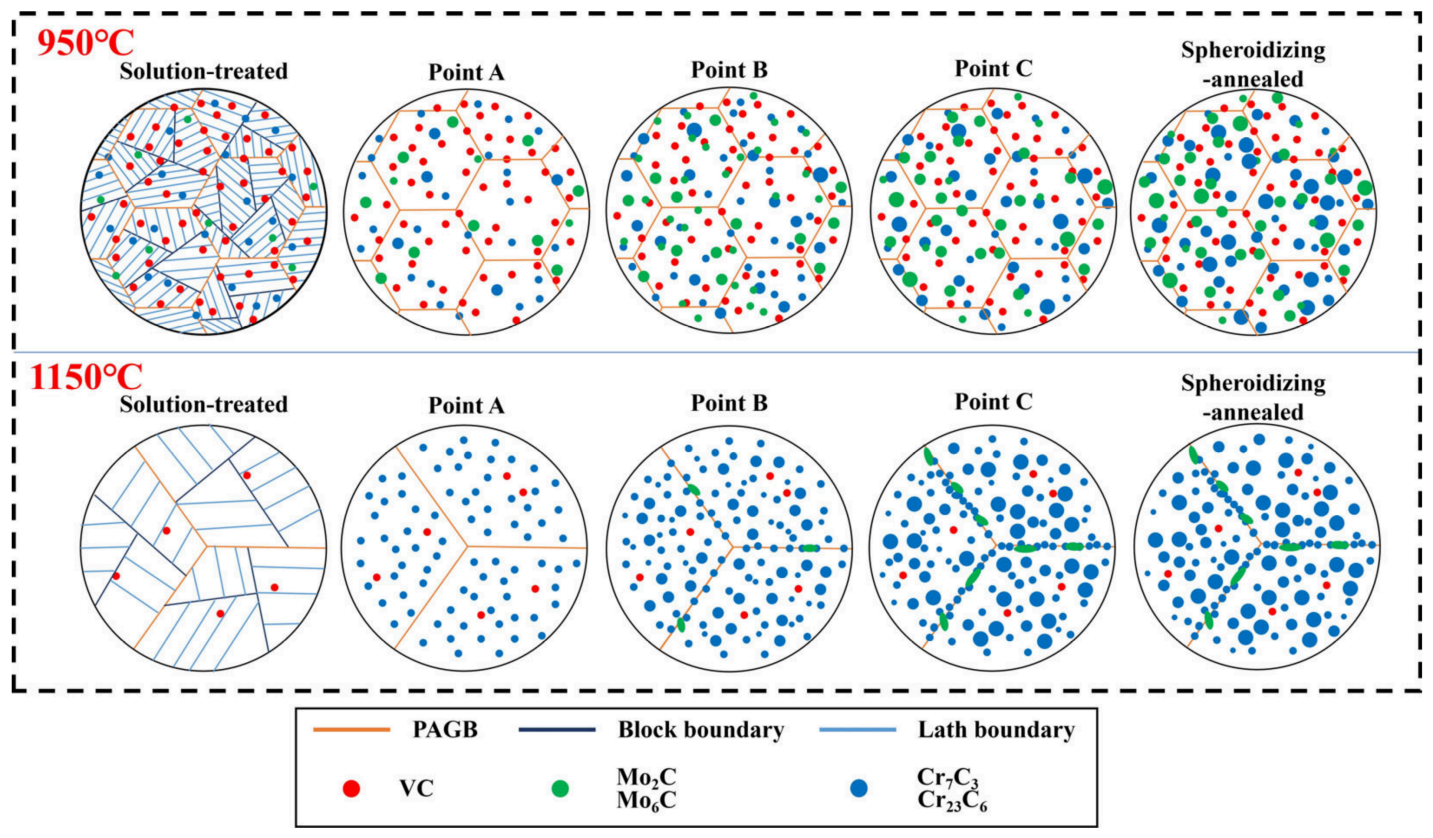
Schematic diagram of Mo-rich carbide evolution during post-forging heat treatment.

**Table 1:** Chemical composition of test steel (wt%).

C	Si	Mn	Cr	Mo	V	Fe
0.37	0.83	0.44	5.22	1.46	0.81	Bal.

**Table 2:** Experimental matrix of the samples.

Sample Designation	Solution Treatment (1 h, Oil Quenching)	Spheroidizing Annealing State
950-ST	950 °C	As-solution-treated
950-A/B/C	950 °C	Interrupted at points A, B, and C of spheroidizing annealing (Figure 1)
950-F	950 °C	Complete spheroidizing annealing
1000-ST	1000 °C	As-solution-treated
1000-F	1000 °C	Complete spheroidizing annealing
1050-ST	1050 °C	As-solution-treated
1050-A/B/C	1050 °C	Interrupted at points A, B, and C of spheroidizing annealing (Figure 1)
1050-F	1050 °C	Complete spheroidizing annealing
1100-ST	1100 °C	As-solution-treated
1100-F	1100 °C	Complete spheroidizing annealing
1150-ST	1150 °C	As-solution-treated
1150-A/B/C	1150 °C	Interrupted at points A, B, and C of spheroidizing annealing (Figure 1)
1150-F	1150 °C	Complete spheroidizing annealing

**Table 3:** V-notch Charpy impact energy and Rockwell hardness of the experimental steel after solution treatment at different temperatures, spheroidizing annealing, and subsequent quenching and triple tempering.

Solution Temperature (°C)	Rockwell Hardness (HRC)	Impact Energy KV2 (J)	Average Impact Energy (J)
950	45.31, 45.88, 45.78, 45.40, 46.32	14.5, 16.9, 18.4	16.6
1000	45.35, 45.74, 45.78, 45.73, 45.32	17.7, 18.4, 18.8	18.3
1050	45.32, 45.02, 44.84, 45.89, 44.87	17.0, 17.9, 18.0	17.6
1100	45.20, 45.14, 44.87, 44.92, 44.90	13.1, 14.8, 16.5	14.8
1150	45.92, 45.35, 45.61, 45.21, 45.86	9.0, 13.6, 16.5	13.0

## Data Availability

The original contributions presented in this study are included in the article/Supplementary Material. Further inquiries can be directed to the corresponding author.

## Supplementary Materials

The following supporting information can be downloaded at: https://www.mdpi.com/article/10.3390/ma19214621/s1, Figure S1: Size distribution of all carbides in the experimental steel at different solution temperatures: (a) ST and (b) F conditions; size statistics of all carbides in the experimental steel at different solution temperatures: (c) area fraction evolution, (d) number density evolution; (e) average size and *d*_max_/*d*_min_ ratio of all carbides.
