# Supplementary material for: Evolution of Mo-Rich Carbides in H13 Hot-Working Die Steel During Spheroidizing Annealing
Source: Materials (Basel). 2026 Oct 30;19(21):4621. doi: 10.3390/ma19214621 (PMC13765112; doi:10.3390/ma19214621)
Supplement: Supplementary file 1 [file materials-19-04621-s001.zip › materials-4616846-supplementary.pdf]

Supplementary Material

for

Evolution of Mo-rich Carbides in H13 Hot-working Die Steel during Spheroidizing Annealing

The size distribution of all carbides in the experimental steel under different solution conditions during post-forging heat treatment with the corresponding statistical results is shown in **Fig. S1**. As the solution temperature increased, the number density of all carbides after spheroidizing annealing increased, while the carbide size remained essentially unchanged. The average size of all carbides decreased slightly in the 1100 °C and 1150 °C solution-treated + spheroidizing-annealed samples.

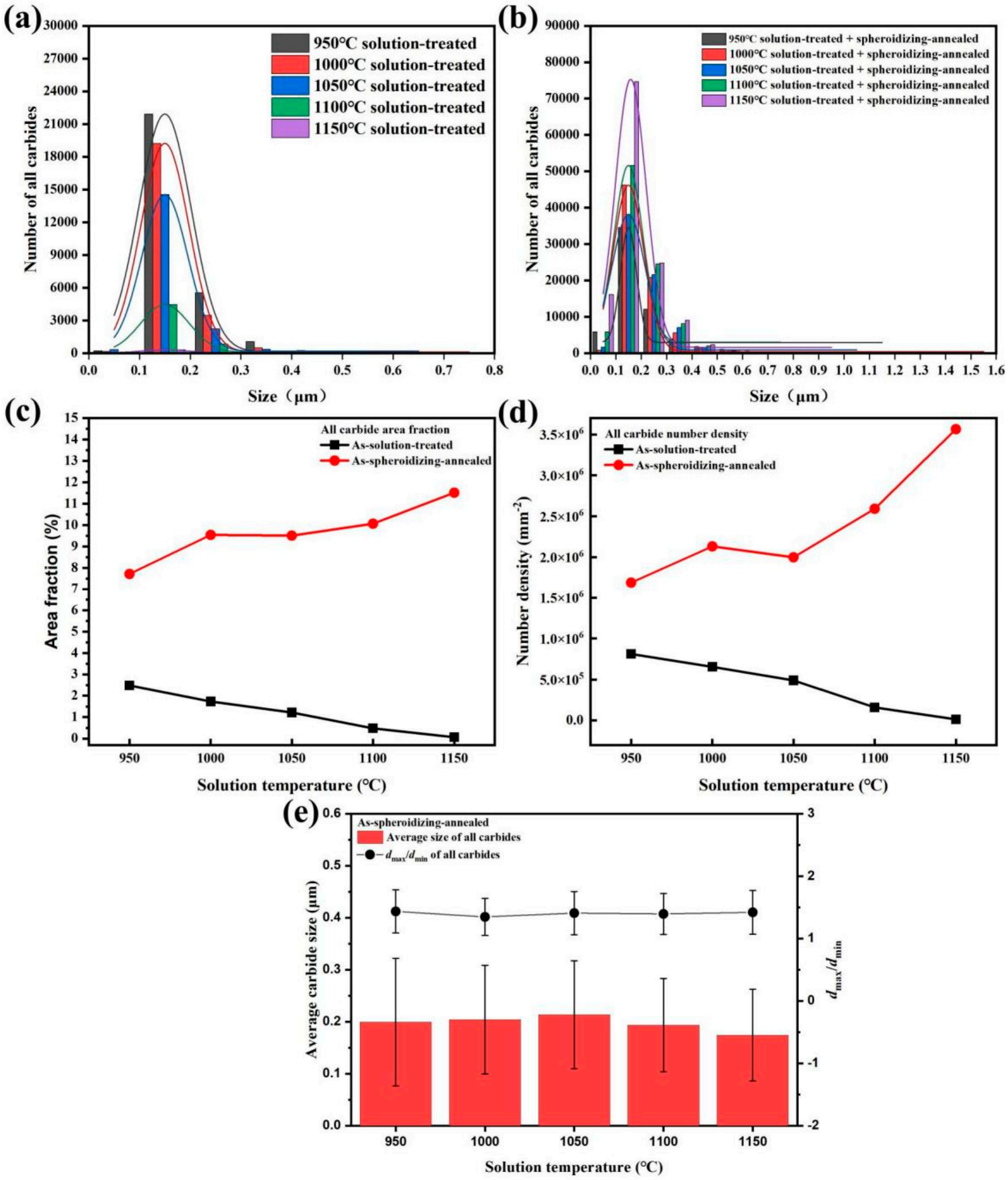

Figure S1. Size distribution of all carbides in the experimental steel at different solution temperatures: (a) ST and (b) F conditions; size statistics of all carbides in the experimental steel at different solution temperatures. (c) Area fraction evolution; (d) number density evolution; (e) average size and dmax/dmin ratio of all carbides.
